# Cytomegalovirus Antiviral Resistance Among Kidney Transplant Recipients in a Phase 3 Trial of Letermovir vs Valganciclovir Prophylaxis

**DOI:** 10.1093/infdis/jiae287

**Published:** 2024-06-10

**Authors:** Julie M Strizki, Tracy L Diamond, Valerie L Teal, Christopher L Gilbert, Weiwen Wang, Nicole Stauffer, Barbara A Haber

**Affiliations:** Merck Research Laboratories, Merck & Co., Inc., Rahway, NJ, USA; Merck Research Laboratories, Merck & Co., Inc., Rahway, NJ, USA; Merck Research Laboratories, Merck & Co., Inc., Rahway, NJ, USA; Merck Research Laboratories, Merck & Co., Inc., Rahway, NJ, USA; Merck Research Laboratories, Merck & Co., Inc., Rahway, NJ, USA; Merck Research Laboratories, Merck & Co., Inc., Rahway, NJ, USA; Merck Research Laboratories, Merck & Co., Inc., Rahway, NJ, USA

**Keywords:** cytomegalovirus, kidney transplant recipient, letermovir, prophylaxis, resistance

## Abstract

**Background:**

In a phase 3 trial, letermovir was noninferior to valganciclovir for cytomegalovirus (CMV) disease prophylaxis in kidney transplant recipients who were CMV-seronegative and received kidneys from donors who were CMV-seropositive. Genotypic antiviral resistance and CMV glycoprotein B (gB) genotype are reported.

**Methods:**

Plasma samples with detectable CMV DNA were sequenced for the presence of known letermovir and valganciclovir resistance-associated amino acid substitutions (RASs) encoded by CMV gene regions (*UL51*, *UL54*, *UL56*, *UL89*, *UL97*) and prevalence of gB (*UL55*) genotypes (gB1–gB5).

**Results:**

Among participants, 84 of 292 (letermovir) and 93 of 297 (valganciclovir) had evaluable data for ≥1 gene target. Letermovir RASs were not detected in participants who received letermovir prophylaxis; however, 3 had valganciclovir RASs (pUL97). Twelve participants who received valganciclovir prophylaxis had valganciclovir RASs (pUL54, pUL97), and 1 who did not receive letermovir during the trial had letermovir RASs (pUL56). All but 1 participant responded to valganciclovir treatment irrespective of breakthrough CMV DNAemia or frequency of RASs. gB1 was the most frequent genotype across all participants and subgroups.

**Conclusions:**

Letermovir RASs were not detected with letermovir prophylaxis, supporting a low risk for development of resistance in kidney transplant recipients who were CMV-seronegative and received kidneys from donors who were CMV-seropositive.

**Clinical Trials Registration:**

ClinicalTrials.gov, NCT03443869; EudraCT, 2017-001055-30.

## Teaser

In a trial of cytomegalovirus prophylaxis with letermovir or valganciclovir for up to 200 days in 589 cytomegalovirus-seronegative kidney transplant recipients who received kidneys from cytomegalovirus-seropositive donors, there was a low risk for development of letermovir resistance.

Cytomegalovirus (CMV) is an opportunistic pathogen that primarily affects individuals who are immunocompromised, including kidney transplant recipients (KTRs). Among KTRs, those who are CMV-seronegative (R−) and receive kidneys from donors who are CMV-seropositive (D+) are at highest risk for developing CMV disease. Valganciclovir has been the standard of care for posttransplant prophylaxis and treatment of CMV disease in KTRs who are CMV D+R− [1, 2].

The US Food and Drug Administration approved letermovir, a selective CMV antiviral, in 2023 for prophylaxis of CMV disease in adult KTRs at high risk (CMV D+R−). Approval was based on results from a phase 3 randomized double-blind trial, which demonstrated that letermovir prophylaxis was noninferior to valganciclovir prophylaxis for the prevention of CMV disease, with lower rates of leukopenia or neutropenia than valganciclovir [3, 4]. The mechanism of action of letermovir—namely, inhibition of CMV terminase—is unique from other CMV antivirals (eg, cidofovir, foscarnet, ganciclovir/valganciclovir, maribavir) whose efficacy can be influenced by resistance-associated amino acid substitutions (RASs) predominantly within CMV DNA polymerase and/or CMV protein kinase subunits pUL54 and pUL97 [5–7]. Letermovir RASs primarily map to the CMV terminase subunit pUL56, although additional substitutions identified in other terminase subunits, including pUL51 and pUL89, may affect letermovir activity [5, 8–10].

CMV glycoprotein B (gB), encoded by the CMV *UL55* gene, is important for viral membrane fusion and is a major target of neutralizing antibodies [11]. Several major genotypes of CMV gB have been identified (gB1–gB5) based on genetic variability of the *UL55* coding sequence [12, 13]. Ganciclovir and letermovir have consistent anti-CMV activity across gB genotypes [14]. The impact of gB genotype on virologic or clinical outcomes in individuals who are immunocompromised, including KTRs, is unclear, with some studies reporting no correlation and others suggesting the opposite [15–23].

Monitoring for the presence of antiviral resistance and determining CMV gB genotype in KTRs with CMV DNAemia is not currently routine in clinical practice. Therefore, the phase 3 randomized double-blind trial comparing letermovir and valganciclovir for CMV prophylaxis provided the opportunity to systematically evaluate specific gene targets in KTRs at high risk (CMV D+R−). The objective of this analysis was to characterize antiviral resistance and gB genotype based on sequencing of the gene-coding regions for *UL51*, *UL54*, *UL55*, *UL56*, *UL89*, and *UL97* from plasma samples with detectable CMV DNA collected from participants evaluated for suspected CMV infection and disease or at the discretion of investigators.

## METHODS

### Participants and Sampling

This phase 3 double-blind multicenter trial (protocol MK-8228-002) based on a randomized controlled design compared 28 weeks (up to 200 days) of CMV disease prophylaxis with letermovir vs valganciclovir in KTRs who were CMV D+R− through 52 weeks posttransplant. Complete details of the trial were reported by Limaye et al [4]. Briefly, from May 2018 to April 2021, participants from 94 global sites were randomized 1:1 to letermovir (480 mg/d) or valganciclovir (900 mg/d), with stratification by receipt of lymphocyte-depleting induction immunosuppression. The letermovir group received acyclovir (400 mg twice daily) for prophylaxis of herpes simplex and varicella zoster virus and a placebo for valganciclovir. The valganciclovir group received placebos for letermovir and acyclovir. Discontinuation of prophylaxis for a lack of efficacy or adverse events was noted.

Protocol-defined dose modifications for valganciclovir and acyclovir were indicated according to creatinine clearance assessments (Cockcroft-Gault formula), as recommended in guidelines and prescribing information [1, 2, 24, 25]. Valganciclovir doses lower than recommended for prophylaxis or dose interruptions due to adverse events were documented. Participants who had ≥4 valganciclovir dose modifications due to changes in creatinine clearance during prophylaxis were noted, given an observation from another analysis that ≥4 modifications were associated with a higher rate of CMV DNAemia vs <4 modifications [data on file].

Upon suspicion of CMV infection or disease based on clinical signs and symptoms or upon discontinuation of prophylaxis with the intent to start CMV treatment, investigators obtained plasma samples for analysis of CMV DNA at a central laboratory (Roche COBAS AmpliPrep/COBAS TaqMan assay; lower limit of quantification, 137 IU/mL), although additional testing may have been performed at local laboratories for clinical decision making. CMV antiviral resistance and gB genotyping were also analyzed from the given plasma samples. Additional plasma samples were collected at 2 prespecified subsequent time points (next scheduled study visit and at week 52) and other time points based on the investigators’ discretion. All investigator-reported cases of CMV disease were assessed by an external independent blinded clinical adjudication committee and confirmed per the diagnostic criteria specified by the CMV Drug Development Forum [26].

### Genotyping for Antiviral Resistance

CMV genotyping was attempted on plasma samples with detectable CMV DNA. The entire coding regions for the CMV genes associated with resistance to letermovir (*UL51*, *UL56*, *UL89*) and valganciclovir (*UL54*, *UL97*) were polymerase chain reaction (PCR) amplified from viral DNA and sequenced by next-generation sequencing (Viroclinics-DDL). The deduced sequences were aligned with a CMV reference strain (GenBank accession CMV_ Merlin_NC006273). Previously characterized resistance-associated mutations (reported as the encoded RASs) for letermovir (Supplementary Table 1) and valganciclovir, detected at a frequency ≥5% of the sequence reads, were reported for samples with evaluable next-generation sequencing data [8, 27–31]. A frequency <20% of the sequence reads was considered low and less likely to be clinically relevant, according to previous reports demonstrating that low-frequency sequence variants were often not reproduceable upon sample retesting [8, 32, 33]. Resistance-associated mutations were deemed treatment emergent (TE) when samples from multiple time points were genotyped from the same participant and the mutation (or mutations) was not detected in an earlier sample. Detected resistance-associated mutations were presented as the encoded RASs.

### Genotyping for CMV gB

DNA extracted from the plasma samples was used for CMV *UL55* gB genotypic testing. To perform gB genotyping, a portion of the *UL55* gene was amplified by PCR from the total DNA isolated. The DNA sequence of the PCR products was compared with those of known gB *UL55* reference sequences to assign a specific gB genotype for each sample (Supplementary Table 2).

### Human Subjects

The study was conducted in accordance with the International Council for Harmonization principles of Good Clinical Practice and was approved by the appropriate institutional review boards and regulatory agencies. All participants gave written informed consent before any study procedures were performed.

## RESULTS

Overall, 589 participants received ≥1 dose of the study regimen. Baseline demographics and characteristics were well balanced across the prophylaxis groups and have been described elsewhere [4]. Among 292 participants in the letermovir group, 84 had genotyping performed, and 67 had evaluable data for at least 1 of the 5 resistance-associated CMV gene-coding regions (*UL51*, *UL54*, *UL56*, *UL89*, or *UL97*). In the valganciclovir group, 93 of 297 participants had genotyping performed, and 82 had evaluable resistance data available for at least 1 of the 5 resistance-associated CMV gene-coding regions (Supplementary Figure 1).

A total of 15 participants had valganciclovir RASs observed in pUL54 and/or pUL97: 3 in the letermovir group and 12 in the valganciclovir group (Table 1). All 15 received treatment for CMV, 7 had confirmed CMV disease, 7 had CMV DNA detected during prophylaxis, and 3 had TE RASs observed (Table 2). Of the 12 participants in the valganciclovir group, 4 discontinued prophylaxis due to a lack of efficacy (V2, V9, V11, V12). All but 1 of the 12 participants in the valganciclovir group (V11) received recommended prophylaxis doses of valganciclovir based on creatinine clearance. One participant in the valganciclovir group (V12), who did not receive letermovir during the trial, had low-frequency letermovir RASs in pUL56 that were not confirmed upon repeat testing.

**Table 1.** Resistance-Associated Amino Acid Substitutions (≥5%)

	Letermovir		Valganciclovir		
Participants	No. (%)	Allele Frequency, %	No. (%)	Allele Frequency, %	Participant
With genotyping performed	84	…	93	…	…
With evaluable resistance data	67	…	82	…	…
With any known letermovir RASs	0 (0.0)	…	1 (1.2)	…	…
pUL51	0 (0.0)	…	0 (0.0)	…	…
pUL56	0 (0.0)	…	1 (1.2)	…	…
F261L	0 (0.0)	…	1 (1.2)	6.8	V12
R369S	0 (0.0)	…	1 (1.2)	8.5	V12
pUL89	0 (0.0)	…	0 (0.0)	…	…
With any known valganciclovir RASs	3 (4.5)	…	12 (14.6)	…	…
pUL54	0 (0.0)	…	4 (4.9)	…	…
F412S	0 (0.0)	…	1 (1.2)	7.1	V10
I726T	0 (0.0)	…	1 (1.2)	11.7	V5
N408K	0 (0.0)	…	1 (1.2)	52.1	V2
Q783R	0 (0.0)	…	1 (1.2)	84.5	V3
V812L	0 (0.0)	…	1 (1.2)	20.5	V2
pUL97	3 (4.5)	…	9 (11.0)	…	…
A594T	1 (1.5)	99.9	0 (0.0)	…	L3
A594V	0 (0.0)	…	3 (3.7)	6.2	V7
	…	…	…	94.2	V10
	…	…	…	99.9	V8
C592G	0 (0.0)	…	1 (1.2)	>98^a^	V9
C603W	0 (0.0)	…	1 (1.2)	6.5	V10
L405P	1 (1.5)	6.3	0 (0.0)	…	L2
L595S	0 (0.0)	…	1 (1.2)	95.0	V11
M460I	1 (1.5)	6.0	5 (6.1)	5.4	L1, V1
	…	…	…	6.4	V6
	…	…	…	11.3	V9
	…	…	…	5.1	V4
	…	…	…	5.3	V12

Abbreviation: RAS, resistance-associated amino acid substitution.

^a^RAS identified at 2 time points.

**Table 2.** Clinical Characteristics of Participants With RASs

		Study Days			CMV			
Prophylaxis: Participant	Age, y; Sex	End of Prophylaxis	First Quantifiable CMV DNA by Central Laboratory	Samples Taken for Genotyping With Detected RASs	Infection Only or Confirmed Disease^a^	Treatment and Duration, Study Days	gB Genotype	Additional Comments
Letermovir								
L1	38 M	199	246	255	Confirmed CMV disease (syndrome)	VGCV 250–360	gB1	NA
L2	45 M	200	299	319	Confirmed CMV disease (syndrome)	VGCV 294–419	gB5	NA
L3	44 M	201	254	355	CMV infection only	VGCV 239–355	gB2	NA
VGCV								
V1	41 M	197	170	203	CMV infection only	VCGV 203–250 and foscarnet 250–251	gB1	CMV DNAemia during prophylaxis
V2	72 F	187	138	232	CMV infection only; CMV infection only	VGCV 187–210; VGCV 231–390	gB4	CMV DNAemia during prophylaxis; prophylaxis discontinued due to lack of efficacy^b^; TE RASs
V3	42 M	195	229	238	CMV infection only	VGCV 235–358	gB3	NA
V4	61 M	196	232	253	Confirmed CMV disease (syndrome)	GCV 252–258 and VGCV 258–385	gB1	NA
V5	61 M	205	226	268	Confirmed CMV disease (syndrome)	VGCV 228–250	gB3	TE RAS
V6	39 M	197	253	270	CMV infection only	GCV 270–292	gB1	NA
V7	66 M	193	250	271	CMV infection only	VGCV 271–313	gB3	NA
V8	41 M	203	237	371	Confirmed CMV disease (syndrome)	VGCV 243–250, GCV 251–259, then VGCV 260–291	gB1	Secondary VGCV prophylaxis on study days 292–371; TE RAS
V9	50 M	145	141	145; 214	CMV infection only; confirmed CMV disease (syndrome)	VGCV 147–154 and GCV 154–176; GCV 226–256	gB5	CMV DNAemia during prophylaxis; prophylaxis discontinued due to lack of efficacy
V10	61 M	153	114	154	CMV infection only	VGCV 154–161, GCV 161, foscarnet 162, then letermovir 163	gB1	CMV DNAemia during prophylaxis; prophylaxis discontinued due to adverse event (visual hallucinations on study day 153); death (myocardial infarction on study day 164)
V11	66 F	92	29	97	Confirmed CMV disease (syndrome)	VGCV 93–198	gB1	CMV DNAemia during prophylaxis; prophylaxis discontinued due to lack of efficacy
V12	42 M	66	59	71	CMV infection only	VGCV 68–120	gB1	CMV DNAemia during prophylaxis; prophylaxis discontinued due to lack of efficacy

Letermovir, given concomitantly with acyclovir; valganciclovir, given concomitantly with a placebo to acyclovir.

Abbreviations: CMV, cytomegalovirus; F, female; gB, glycoprotein B; GCV, ganciclovir; M, male; NA, not applicable; RAS, resistance-associated amino acid substitution; TE, treatment emergent; VGCV, valganciclovir.

^a^Some participants had more than 1 CMV event. CMV disease was confirmed by the independent blinded adjudication committee.

^b^Prophylaxis was discontinued near completion (within 2 weeks) due to lack of efficacy based on a local laboratory result.

**Figure 1.**
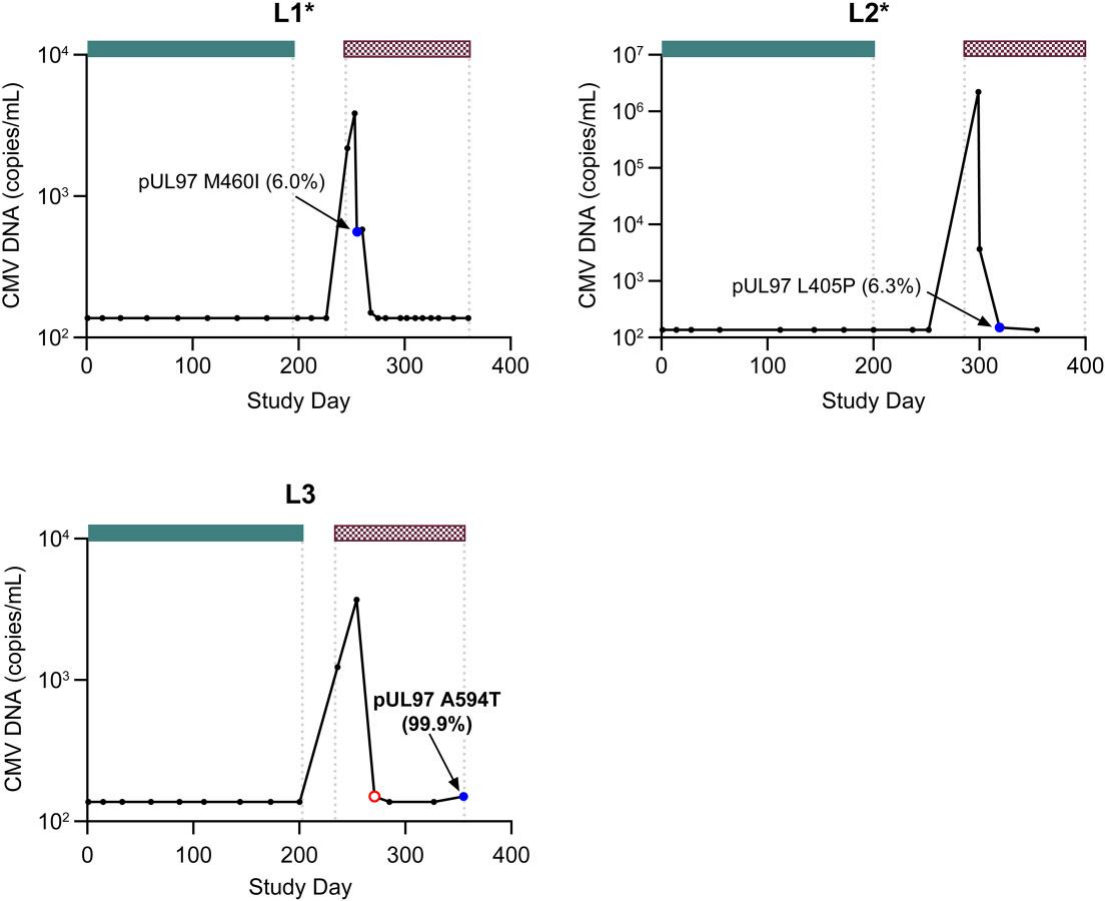
Cytomegalovirus (CMV) DNA profiles and resistance-associated amino acid substitutions (RASs) in participants who completed letermovir prophylaxis. Bars along the top represent CMV antivirals, and the length of each bar indicates the duration; gaps reflect periods when CMV antivirals were not administered. Text labels adjacent to selected symbols indicate specific RASs; the percentage represents the frequency of next-generation sequencing reads in the CMV DNA sequence encoding the RAS, with percentages ≥20% bolded. 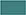 letermovir prophylaxis; 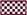 valganciclovir treatment. Symbols reflect the outcome from genotypic analysis: 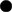 sample not collected; 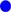 RAS detected; 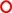 sequencing failed. *Confirmed CMV disease.

Letermovir RASs were not detected in any one receiving letermovir prophylaxis (Table 1). Three participants in the letermovir group (L1–L3) had valganciclovir RASs in pUL97 detected after valganciclovir treatment was initiated (Figure 1). CMV DNAemia occurred 47, 99, and 35 days after completion of prophylaxis for L1, L2, and L3, respectively (Table 2). For L1 and L2, CMV DNA fell below the lower limit of quantification 2.5 to 3.5 weeks after initiation of valganciclovir treatment in the presence of low-frequency valganciclovir RASs in pUL97, and CMV DNA remained suppressed through the remainder of 16 to 18 weeks of treatment. L3's CMV DNA declined 4.5 weeks after the start of treatment with valganciclovir. A high-frequency valganciclovir RAS in pUL97 was detected at the last study visit and after 16.5 weeks of treatment.

**Figure 2.**
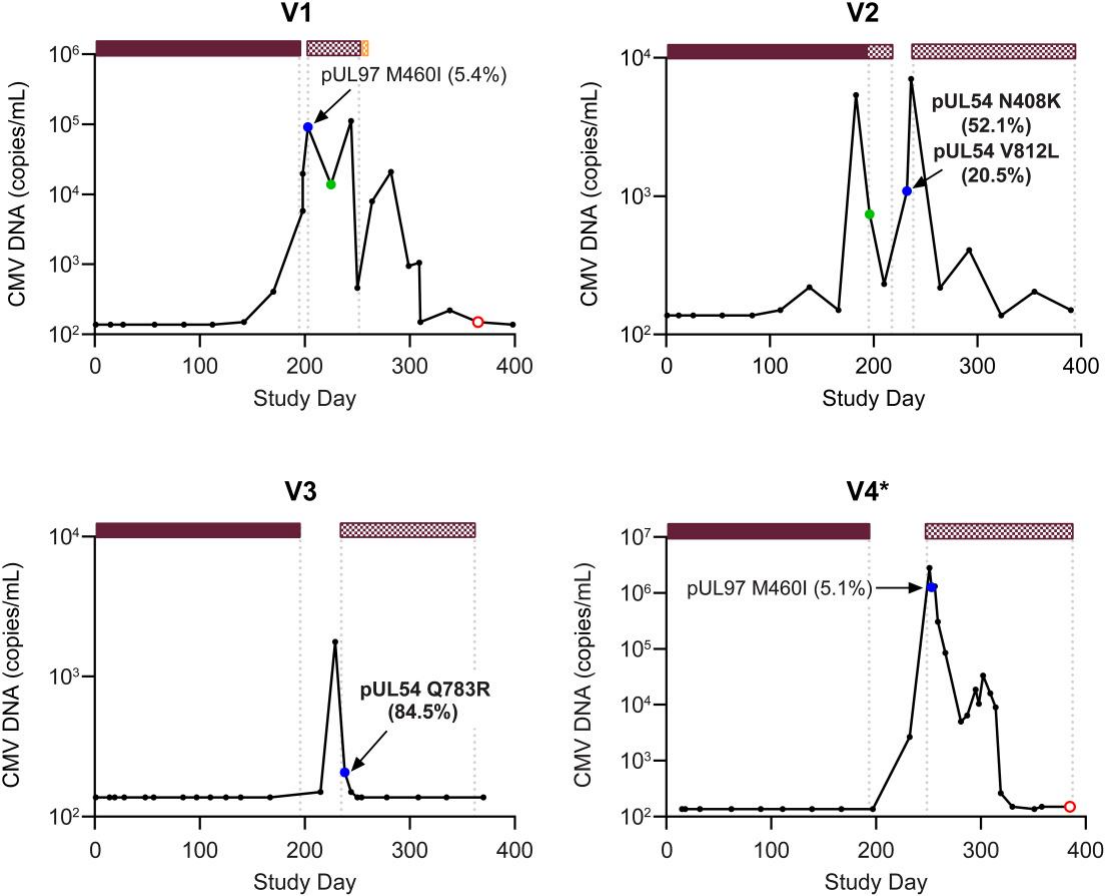
Cytomegalovirus (CMV) DNA profiles in participants with resistance-associated amino acid substitutions (RASs) detected early (within 2 months) after or near completion of valganciclovir prophylaxis. Bars along the top represent CMV antivirals, and the length of each bar indicates the duration; gaps reflect periods when CMV antivirals were not administered. Text labels adjacent to selected symbols indicate specific RASs; the percentage represents the frequency of next-generation sequencing reads in the CMV DNA sequence encoding the RAS, with percentages ≥20% bolded. 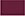 valganciclovir prophylaxis; 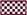 valganciclovir/ganciclovir treatment; 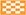 foscarnet treatment. Symbols reflect the outcome from genotypic analysis: 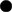 sample not collected; 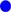 RAS detected; 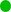 RAS not detected; 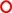 sequencing failed. *Confirmed CMV disease.

Valganciclovir RASs were detected early (within 2 months) after or near completion of valganciclovir prophylaxis in V1 to V4 (Figure 2). V1 and V4 had low-frequency RASs detected in pUL97, and V2 and V3 had high-frequency RASs detected in pUL54. V1 developed CMV DNAemia during prophylaxis and was treated for 7 weeks with valganciclovir and 2 days of foscarnet. A low-frequency RAS in pUL97 was detected 6 days after completion of prophylaxis. V2 had 5 valganciclovir dose modifications based on creatinine clearance changes and developed CMV DNAemia during prophylaxis. Upon discontinuation of prophylaxis, the participant received 3 weeks of valganciclovir treatment for asymptomatic primary CMV infection. High-frequency TE RASs in pUL54 were detected 3 weeks after valganciclovir treatment was stopped. V3 developed CMV DNAemia 34 days after completion of prophylaxis and had a high-frequency RAS in pUL54 detected 3 days after initiating valganciclovir treatment. V4 had 7 valganciclovir dose modifications based on creatinine clearance changes, developed CMV DNAemia 36 days after completion of prophylaxis, and had a low-frequency RAS in pUL97 detected 57 days from completion of prophylaxis. Ganciclovir treatment was started the day before the RAS was detected.

**Figure 3.**
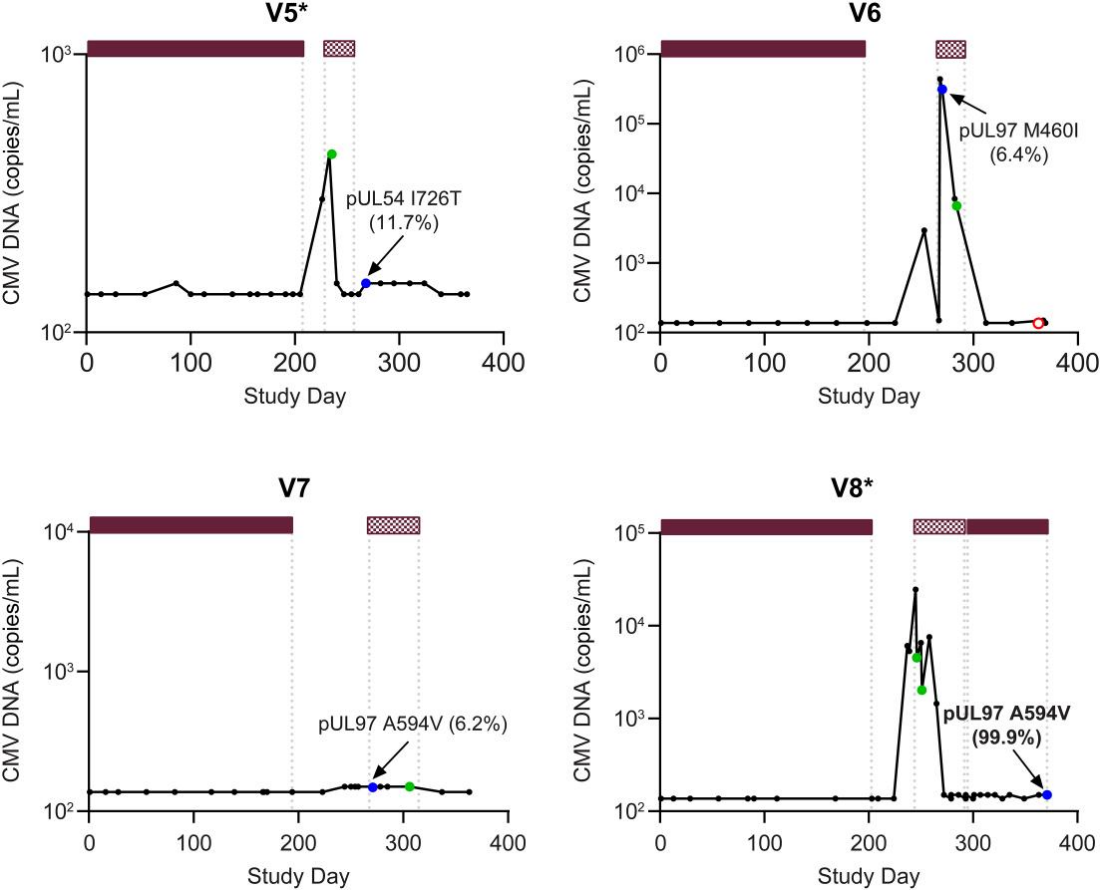
Cytomegalovirus (CMV) DNA profiles in participants with resistance-associated amino acid substitutions (RASs) detected later (>2 months) after completion of valganciclovir prophylaxis. Bars along the top represent CMV antivirals, and the length of each bar indicates the duration; gaps reflect periods when CMV antivirals were not administered. Text labels adjacent to selected symbols indicate specific RASs; the percentage represents the frequency of next-generation sequencing reads in the CMV DNA sequence encoding the RAS, with percentages ≥20% bolded. 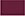 valganciclovir prophylaxis; 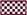 valganciclovir/ganciclovir treatment. Symbols reflect the outcome from genotypic analysis: 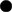 sample not collected; 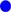 RAS detected; 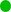 RAS not detected; 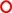 sequencing failed. *Confirmed CMV disease.

Valganciclovir RASs were detected later (>2 months) postprophylaxis in V5 to V8 (Figure 3). V5 developed CMV DNAemia 21 days after completion of prophylaxis. A low-frequency TE RAS in pUL54 was detected 18 days after valganciclovir treatment ended. V6 had 4 valganciclovir dose modifications based on creatinine clearance changes and an interruption in dosing due to leukopenia. This participant developed CMV DNAemia 56 days after prophylaxis completion, and a low-frequency RAS in pUL97 was detected 73 days from prophylaxis completion (ganciclovir treatment began on the same day). V7 had 8 valganciclovir dose modifications for creatinine clearance changes and polyomavirus (BK virus)–associated nephropathy. CMV DNAemia occurred 51 days after completion of prophylaxis, and a low-frequency RAS in pUL97 was detected 78 days after completion of prophylaxis (valganciclovir treatment began on the same day). V8 developed CMV DNAemia 34 days after prophylaxis ended. Valganciclovir/ganciclovir treatment started on study day 243 (6 days after CMV DNAemia occurred) and continued for 7 weeks. The participant received secondary valganciclovir prophylaxis on study days 292 through 371. A high-frequency TE RAS in pUL97 was detected on study day 371.

**Figure 4.**
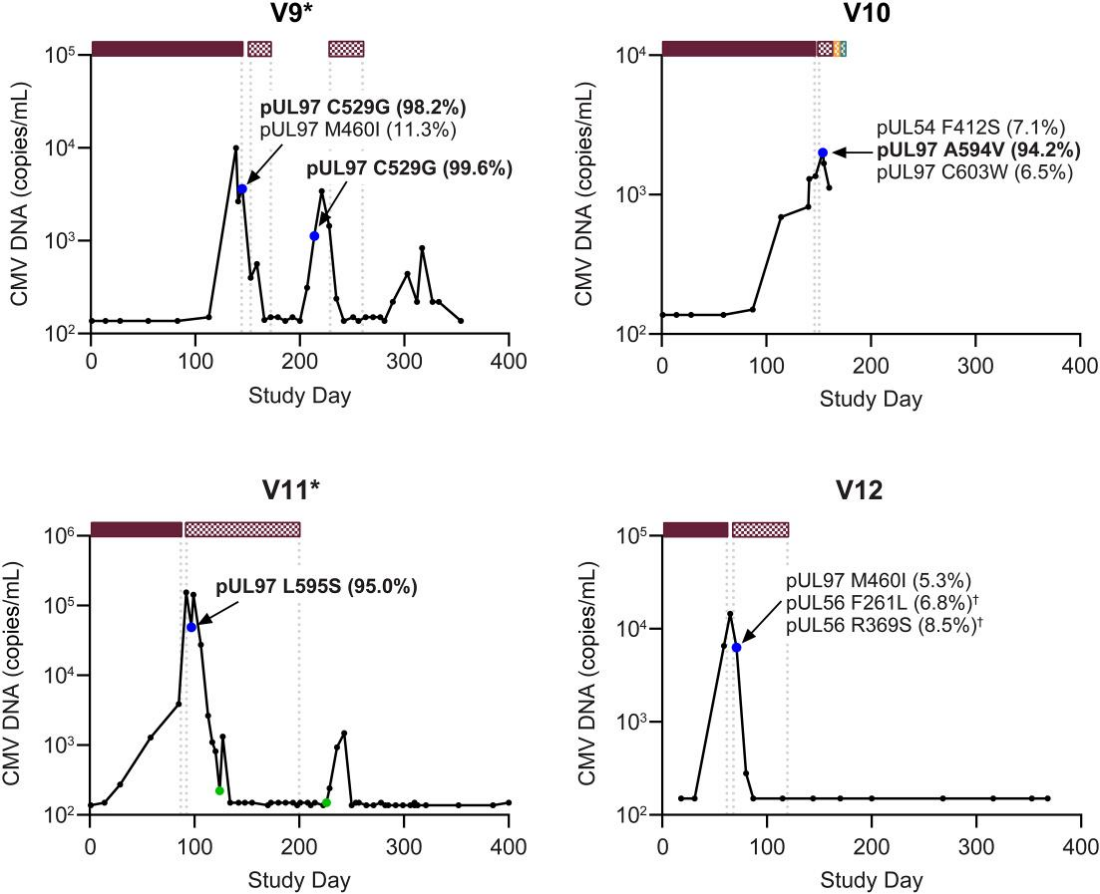
Cytomegalovirus (CMV) DNA profiles and resistance-associated amino acid substitutions (RASs) detected in participants who did not complete valganciclovir prophylaxis. Bars along the top represent CMV antivirals, and the length of each bar indicates the duration; gaps reflect periods when CMV antivirals were not administered. Text labels adjacent to selected symbols indicate specific RASs; the percentage represents the frequency of next-generation sequencing reads in the CMV DNA sequence encoding the RAS, with percentages ≥20% bolded. 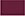 valganciclovir prophylaxis; 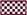 valganciclovir/ganciclovir treatment; 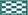 letermovir treatment; 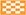 foscarnet treatment. Symbols reflect the outcome from genotypic analysis: 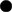 sample not collected; 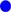 RAS detected; 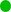 RAS not detected. *Confirmed CMV disease. ^†^RAS not detected upon repeat next-generation sequencing.

Four participants (V9–V12) did not complete valganciclovir prophylaxis, and all 4 developed CMV DNAemia during prophylaxis (Figure 4). V9 developed CMV DNAemia on study day 113. High- and low-frequency RASs in pUL97 were detected on the same day that prophylaxis was discontinued (study day 145) due to a lack of efficacy. The participant was treated with valganciclovir/ganciclovir for 4 weeks. Shortly thereafter, CMV DNA increased again, and the same high-frequency RAS in pUL97 was detected again. V10 had 7 dose modifications based on creatinine clearance changes and stopped prophylaxis on study day 153 due to an adverse event of visual hallucination unrelated to the study drug. Prophylaxis was discontinued, treatment for CMV with valganciclovir was started the next day, and high- and low-frequency RASs in pUL97 and pUL54 were detected on that same day. After 6 days of valganciclovir treatment, the participant was hospitalized for worsening hallucinations (likely metabolic etiology) and switched to ganciclovir, foscarnet, and letermovir (each for 1 day consecutively) before dying on study day 164 due to myocardial infarction and cardiopulmonary arrest unrelated to the study drug. V11 received a lower-than-recommended valganciclovir dose based on creatinine clearance for 3 weeks of the prophylaxis period, developed CMV DNAemia, discontinued prophylaxis due to a lack of efficacy (study day 92), and switched to valganciclovir treatment for 15 weeks. A high-frequency RAS in pUL97 was detected on day 4 of valganciclovir treatment. V12 developed CMV DNAemia on day 59 of prophylaxis. The participant discontinued prophylaxis for a lack of efficacy on study day 66, and treatment with valganciclovir was initiated 2 days later. On day 3 of valganciclovir treatment, low-frequency RASs were detected in pUL97 and pUL56. This participant did not receive letermovir during the study, and upon repeat sequencing of *UL56* from the same sample, the letermovir RASs were not detected.

The exploratory analysis of CMV *UL55* identified that the distribution of gB genotypes was generally comparable across the letermovir and valganciclovir groups. In both groups, gB1 was the most frequent genotype (50.8% and 36.3%, respectively), followed by gB3 (21.5% and 31.3%). Among those with confirmed CMV disease, gB1 was also the most frequent genotype (41.7% and 43.3%), followed by gB3 (29.2% and 30.0%; Table 3). Of the 3 participants in the letermovir group who had valganciclovir RASs, each had a unique gB genotype (gB1, gB5, and gB2). gB1 was the most frequent genotype (7 participants) among the 12 participants with RASs in the valganciclovir group (Table 2).

**Table 3.** Prevalence of CMV gB (*UL55*) Genotypes

Participants	Letermovir	Valganciclovir
With genotyping performed	84	93
With evaluable gB data	65	80
gB1	33 (50.8)	29 (36.3)
gB2	8 (12.3)	15 (18.8)
gB3	14 (21.5)	25 (31.3)
gB4	8 (12.3)	10 (12.5)
gB5	2 (3.1)	1 (1.3)
With genotyping performed and CMV disease^a^	25^b^	31^c^
With evaluable gB data	24	30
gB1	10 (41.7)	13 (43.3)
gB2	4 (16.7)	3 (10.0)
gB3	7 (29.2)	9 (30.0)
gB4	2 (8.3)	4 (13.3)
gB5	1 (4.2)	1 (3.3)

Data are presented as No. (%) of participants with a gB genotype result available. gB genotype (detected ≥90% of mapped reads) was assessed at the first time point with evaluable *UL55* sequence data. Letermovir, given concomitantly with acyclovir; valganciclovir, given concomitantly with a placebo to acyclovir.

Abbreviations: CMV, cytomegalovirus; gB, glycoprotein B.

^a^CMV disease confirmed by the independent blinded adjudication committee.

^b^Five participants in the letermovir group had CMV disease but did not have genotyping performed (CMV disease/early discontinuation sample and resistance sample collected on different dates, n = 3; sample not collected, n = 1; undetectable CMV DNA, n = 1).

^c^Four participants in the valganciclovir group had CMV disease but did not have genotyping performed (sample not collected, n = 2; CMV disease/early discontinuation sample and resistance sample collected on different dates, n = 1; undetectable CMV DNA, n = 1).

## DISCUSSION

In this large phase 3 trial comparing letermovir vs valganciclovir prophylaxis in KTRs who were CMV D+R−, no letermovir RASs were detected among those who received letermovir prophylaxis, and 15 participants had valganciclovir RASs detected: 3 in the letermovir group and 12 in the valganciclovir group. After participants finished prophylaxis (ie, completed the planned duration or discontinued early), valganciclovir RASs were detected in all except 1, who had detectable RASs on the same day that valganciclovir prophylaxis was discontinued. The 3 participants in the letermovir group completed prophylaxis, and valganciclovir RASs were detected while they were receiving valganciclovir treatment. Of the 12 participants in the valganciclovir group, 8 completed prophylaxis, and valganciclovir RASs were detected during or after valganciclovir treatment. The 4 participants in the valganciclovir group who discontinued prophylaxis early had valganciclovir RASs detected 0 to 5 days after prophylaxis was stopped. Three valganciclovir RASs in the valganciclovir group were TE, occurring after or during valganciclovir treatment. One participant who received valganciclovir prophylaxis also had 2 letermovir RASs detected at low frequencies. gB1 and gB3 were the most frequent genotypes across the letermovir and valganciclovir groups in all participants who had evaluable gB data and among those who developed confirmed CMV disease. No trend was identified for gB genotype in participants with RASs.

Letermovir RASs were not detected in any one who received letermovir prophylaxis, supporting that letermovir resistance is rare in the setting of primary prophylaxis in KTRs who are CMV D+R− and at high risk for CMV infection or disease. This has been established with clinical trial and real-world data in cases of allogeneic R+ hematopoietic cell transplantation as well [8, 34, 35]. The 3 participants who received letermovir prophylaxis and had valganciclovir RASs detected responded to treatment with valganciclovir. Of note, CMV variants pUL97 L405P and A594T have <3-fold decreased susceptibility to valganciclovir, while CMV pUL97 M460I has ∼8-fold decreased susceptibility to valganciclovir [36–38]. pUL97 L405P and M460I were present at low frequencies (each ∼6%), and their clinical significance is unknown. Although the frequency for pUL97 A594T was 99.9% in L3, this RAS was detected after prolonged valganciclovir treatment, and there are no data available after this time point to help understand the clinical relevance in this case.

In all but 1 of the 12 participants in the valganciclovir group, valganciclovir RASs were detected after completion or early discontinuation of prophylaxis. Breakthrough CMV DNAemia during valganciclovir prophylaxis, a known risk factor for the development of valganciclovir resistance [39–42], occurred in 7 participants. All but 1 responded to valganciclovir treatment irrespective of breakthrough CMV DNAemia or frequency of RASs, calling into question their clinical relevance.

No letermovir RASs were detected in the letermovir group. However, 1 participant who received valganciclovir prophylaxis and had no exposure to letermovir in the trial had letermovir RASs detected in pUL56. The frequency of the letermovir RASs was low (F261L, 6.8%; R369S, 8.5%), and the clinical significance is unclear. Repeat sequencing of pUL56 from the same sample did not detect any letermovir RASs, indicating that their detection may have been a sequencing artifact.

Although no known RASs for letermovir were detected, novel substitutions at known resistance loci in pUL56 (S229 and M329) and pUL89 (D344) were detected at low frequencies (5%–7%) in the letermovir prophylaxis group (S229Y, n = 1; M329I, n = 9; D344Y, n = 5). Similar numbers in the valganciclovir group (n = 1, 8, and 5, respectively) had these same 3 novel substitutions observed [data on file]. The effects of these novel substitutions on letermovir susceptibility are unknown but anticipated to be minimal as their frequencies were low.

Historically, valganciclovir has been used for prophylaxis and treatment of CMV in KTRs, as there were few antivirals active against CMV [43]. Prior exposure to valganciclovir in the setting of subtherapeutic concentrations (ie, with intentional low dosing or fluctuating kidney function), prolonged duration, or infection onset during prophylaxis increases the risk for valganciclovir resistance [6, 39, 40, 44]. A potential benefit of utilizing letermovir for primary prophylaxis is the distinct mechanism of action of CMV terminase inhibition, thereby preserving use of CMV antivirals that inhibit CMV DNA polymerase and/or CMV kinase for the treatment of CMV [45, 46]. Further exploration is needed to determine if primary letermovir prophylaxis limits the development of resistance to other CMV antivirals in KTRs who are CMV D+R−.

In the current CMV landscape, RASs have been reported for multiple transplant types (eg, stem cell, lung, heart, kidney, liver), variations of CMV serostatus (D+R−, D+R+, D–R+), and different durations of exposures collectively, rather than exclusively, for KTRs who are CMV D+R− [5, 28, 32, 47]. The last report of a systematic assessment of RASs in KTRs who are CMV D+R− was an analysis from the IMPACT trial published in 2012 [7]. The true rate of antiviral resistance in the transplant population is unknown. Routine testing of any observed CMV DNAemia for resistance-associated mutations is not performed and is generally limited to cases of suspected or confirmed refractory/resistant CMV disease [2]. Additionally, next-generation sequencing is an improved approach for resistance testing that is more sensitive in detecting minor variants than Sanger sequencing, which has been traditionally used to detect resistance-associated mutations [27, 28, 48]. gB1 and gB3 have been reported as the most frequent gB genotypes in solid organ transplant recipients [15, 49]. The exploratory analysis in this large population of KTRs had similar findings and did not identify an association between gB genotype and confirmed CMV disease or RASs.

Strengths of this analysis include the following: (1) it is the first description of RASs from a large randomized double-blind trial that compared 2 antivirals approved for CMV prophylaxis for a planned duration of ∼200 days in KTRs who were CMV D+R−, and (2) the evaluation for RASs was performed systematically in those who were treated for CMV after completion or early discontinuation of prophylaxis.

There are 2 notable limitations of this analysis: (1) baseline sequencing was not performed pretransplant on the CMV-seropositive donor kidney or posttransplant on the study participant due to undetectable CMV DNA at the time of randomization, and (2) resistance samples were not collected at regular intervals with CMV DNA samples such that the first signs of resistance may have occurred at an earlier time point than detected (eg, during prophylaxis, prior to the start of treatment). Therefore, it could not be definitively determined if the RASs were transferred from the CMV-seropositive donor kidney or if they emerged during prophylaxis or treatment. Among the 12 participants with RASs in the valganciclovir group, 5 had RASs detected prior to the start of treatment, and the remaining 7 had RASs detected after treatment start (4 were detected within 1–4 days after treatment start). The overall longer exposure to valganciclovir over the course of the trial in the valganciclovir group may have led to the increased detection of RASs in this group as compared with the letermovir group. Of note, it cannot be ruled out that some RASs could have been artifacts of the sequencing method, given the low frequencies at which they occurred. Review of data from other CMV genotyping studies suggest that low-frequency variants (<20%) may be due to sequencing errors [8, 30, 33] and require confirmatory testing. In this study, most participants with valganciclovir RASs detected following prophylaxis responded to valganciclovir treatment. The data available leave unresolved whether some valganciclovir RASs, especially those at low frequency, are due to sequencing errors or are RASs overcome by the higher valganciclovir concentrations achieved with treatment dosing.

In summary, in this large randomized trial, letermovir was effective for CMV prophylaxis in KTRs who were CMV D+R− and was not associated with development of letermovir RASs.

## Supplementary Data

Supplementary materials are available at *The Journal of Infectious Diseases* online (http://jid.oxfordjournals.org/). Supplementary materials consist of data provided by the authors that are published to benefit the reader. The posted materials are not copyedited. The contents of all supplementary data are the sole responsibility of the authors. Questions or messages regarding errors should be addressed to the corresponding authors.

## Supplementary Material

jiae287_Supplementary_Data

## Notes

***Acknowledgments.*** We thank the study participants, their families and caregivers, and all investigators and site personnel. We thank Ernest Asante-Appiah, PhD, an employee of Merck & Co, Inc, for his thoughtful review of the manuscript. Medical writing and editorial assistance were provided by Dorothy McCoy, PharmD, BCPS, BCIDP, and Carol Zecca, BS, from Merck & Co, Inc, Rahway, NJ, USA.

***Author contributions.*** J. M. S., T. L. D., C. L. G., N. S., and B. A. H. designed and/or monitored the study. V. L. T. and W. W. analyzed, assessed, and verified the study data. All authors interpreted the study data, contributed to development of the manuscript, and approved the final version for submission.

***Data sharing.*** The data-sharing policy, including restrictions, of Merck Sharp & Dohme LLC, a subsidiary of Merck & Co, Inc, Rahway, NJ, USA is available at http://engagezone.msd.com/ds_documentation.php. Requests for access to the clinical study data can be submitted through the Engage Zone site or via email to the Data Access mailbox.

***Role of funder.*** Merck Sharp & Dohme LLC, a subsidiary of Merck & Co, Inc, Rahway, NJ, USA funded this study and had a role in the design and conduct of the study; in the management, analysis, and interpretation of the data; and in preparation and review of the manuscript.

***Financial support*.** This work was supported by Merck Sharp & Dohme LLC, a subsidiary of Merck & Co, Inc, Rahway, NJ, USA.
