# Supplementary material for: Cytomegalovirus Antiviral Resistance Among Kidney Transplant Recipients in a Phase 3 Trial of Letermovir vs Valganciclovir Prophylaxis
Source: J Infect Dis. 2024 Jun 10;230(6):e1287–98. doi: 10.1093/infdis/jiae287 (PMC11646593; doi:10.1093/infdis/jiae287)
Supplement: jiae287_Supplementary_Data [file jiae287_supplementary_data.docx]

**Supplemental Online Content**

Strizki JM, Diamond TL, Teal VL, Gilbert CL, Wang W, Stauffer N, Haber BA. Cytomegalovirus antiviral resistance among kidney transplant recipients in a phase 3 trial of letermovir vs valganciclovir prophylaxis.

This supplemental material has been provided by the authors to give readers additional information about their work.

Table S1. Fold shift in letermovir EC_50_ for phenotypically characterized resistance-associated amino acid substitutions in CMV pUL51, pUL56, and pUL89 encoded by *UL51*, *UL56*, and *UL89* genotypic variants

| **Viral Protein** | **RAS** | **Fold shift in EC_50_** | **Reference(s)** |
| --- | --- | --- | --- |
| pUL51^a^ | P91S | 2.1 | 5 |
|  | A95V | 13.8 | 12 |
|  |  |  |  |
| pUL56^b^ | C25F | 5.4 | 6 |
|  | S229F | 1.8 | 5 |
|  | V231A | 2.1 | 3 |
|  | V231L | 5, 5.1 | 3, 7 |
|  | N232Y | 17 | 4 |
|  | V236A | 2.9 | 6 |
|  | V236L | 14 | 3 |
|  | V236M | 22-46 | 3, 7, 8, 9 |
|  | E237D | 10 | 3 |
|  | L241P | 96, 221 | 3, 7 |
|  | T244K | 3.3 | 3 |
|  | T244R | N/a^d^ | 3 |
|  | L254F | 3.2 | 5 |
|  | L257F | 8.6 | 5 |
|  | L257I | 4.9 | 3 |
|  | K258E | 14 | 4 |
|  | F261C | 4.4 | 3 |
|  | F261L | 2.8 | 3 |
|  | F261S | N/a^d^ | 3 |
|  | Y321C | 4.6 | 3 |
|  | C325F | >3000 | 3 |
|  | C325R | >3000 | 3 |
|  | C325W | 9300 | 6 |
|  | C325Y | >3000, 8796 | 3, 7 |
|  | L328V | 1.9 | 6 |
|  | M329T | 4.4 | 3 |
|  | A365S | 2.0 | 6 |
|  | N368D | 2 | 5 |
|  | R369G | 44 | 7 |
|  | R369M | 2 | 7 |
|  | R369S | 66 | 7 |
|  | R369T | 52 | 11 |
|  | R369K | 4.9 | 10 |
|  | Q234R | 2.0 | 10 |
|  | V363I | 3.7 | 10 |
|  |  |  |  |
| pUL89^c^ | N320H | 1.8 | 4 |
|  | D344E | 1.6 | 5 |
|  | M359I | 1.5 | 4 |

Abbreviations: EC_50_, 50% effective concentration; N/a, not available; RAS(s), resistance-associated substitution(s).

^a^Reference letermovir RASs were previously identified by recombinant phenotyping of UL51 mutations detected in CMV strains selected *in vitro* for resistance to letermovir [5, 12].

^b^Reference letermovir RASs were previously identified by recombinant phenotyping of UL56 mutations detected in CMV strains selected *in vitro* for resistance to letermovir [3-9].

^c^Reference letermovir RASs (relative to the pUL89 amino acid sequence from a reference CMV strain) were previously identified by recombinant phenotyping of UL89 mutations detected in CMV strains selected *in vitro* for resistance to letermovir [4, 5].

^d^The pUL56 substitutions T244R and F261S were observed in single instances (Table 1 and Table 2 of [3]); they were not phenotyped but were considered similar to T244K and F261L/C, respectively.

References

1. Lischka P, Zhang D, Holder D, Zimmermann H. Impact of glycoprotein B genotype and naturally occurring ORF UL56 polymorphisms upon susceptibility of clinical human cytomegalovirus isolates to letermovir. Antiviral Res 2016; 132:204-9.
2. Goldner T, Zimmermann H, Lischka P. Phenotypic characterization of two naturally occurring human cytomegalovirus sequence polymorphisms located in a distinct region of ORF UL56 known to be involved in in vitro resistance to letermovir. Antiviral Res 2015; 116:48-50.
3. Chou S. Rapid in vitro evolution of human cytomegalovirus UL56 mutations that confer letermovir resistance. Antimicrob Agents Chemother 2015; 59:6588-93.
4. Chou S. Comparison of cytomegalovirus terminase gene mutations selected after exposure to three distinct inhibitor compounds. Antimicrob Agents Chemother 2017; 61:e01325-17.
5. Chou S. A third component of the human cytomegalovirus terminase complex is involved in letermovir resistance. Antiviral Res 2017; 148:1-4.
6. Chou S, Satterwhite LE, Ercolani RJ. New locus of drug resistance in the human cytomegalovirus UL56 gene revealed by in vitro exposure to letermovir and ganciclovir. Antimicrob Agents Chemother 2018; 62:e00922-18.
7. Goldner T, Hempel C, Ruebsamen-Schaeff H, Zimmermann H, Lischka P. Geno- and phenotypic characterization of human cytomegalovirus mutants selected in vitro after letermovir (AIC246) exposure. Antimicrob Agents Chemother 2014; 58:610-3.
8. Lischka P, Michel D, Zimmermann H. Characterization of cytomegalovirus breakthrough events in a phase 2 prophylaxis trial of Letermovir (AIC246, MK 8228). J Infect Dis 2016; 213:23-30.
9. Piret J, Goyette N, Boivin G. Drug susceptibility and replicative capacity of multidrug-resistant recombinant human cytomegalovirus harboring mutations in UL56 and UL54 Genes. Antimicrob Agents Chemother 2017; 61:e01044-17.
10. Chou S, Kleiboeker S. Relative frequency of cytomegalovirus UL56 gene mutations detected in genotypic letermovir resistance testing. Antiviral Res 2022; 207:105422.
11. Douglas CM, Barnard R, Holder D, Leavitt R, Levitan D, et al. Letermovir resistance analysis in a clinical trial of cytomegalovirus prophylaxis for hematopoietic stem cell transplant recipients. J Infect Dis 2020; 221(7):1117-1126.
12. Muller C, Tilloy V, Frobert E, Feghoul L, Garrigue I, Lepiller Q, Mirand A, Sidorov E, Hantz S, Alain S. First clinical description of letermovir resistance mutation in cytomegalovirus UL51 gene and potential impact on the terminase complex structure. Antiviral Res 2022;204:105361.

Table S2. Reference sequences in CMV *UL55*

| **Genbank ID** | **gB Genotype** | **Isolate** |
| --- | --- | --- |
| NC_006273.2 | 1 | Merlin Strain |
| FJ527563.1 | 2 | AD169 Strain |
| GU937742.2 | 3 | Toledo Strain |
| KR534210.1 | 4 | JER5268 Strain |
| KJ361971.1 | 5 | UKNEQAS1 Strain |

Abbreviation: gB, glycoprotein B.

Provided by DDL Diagnostic Laboratory, Visseringlaan 25, 2288ER Rijswijk, The Netherlands.

Figure S1. Participants with antiviral resistance and glycoprotein B testing in a study of letermovir vs valganciclovir for CMV prophylaxis in kidney transplant recipients


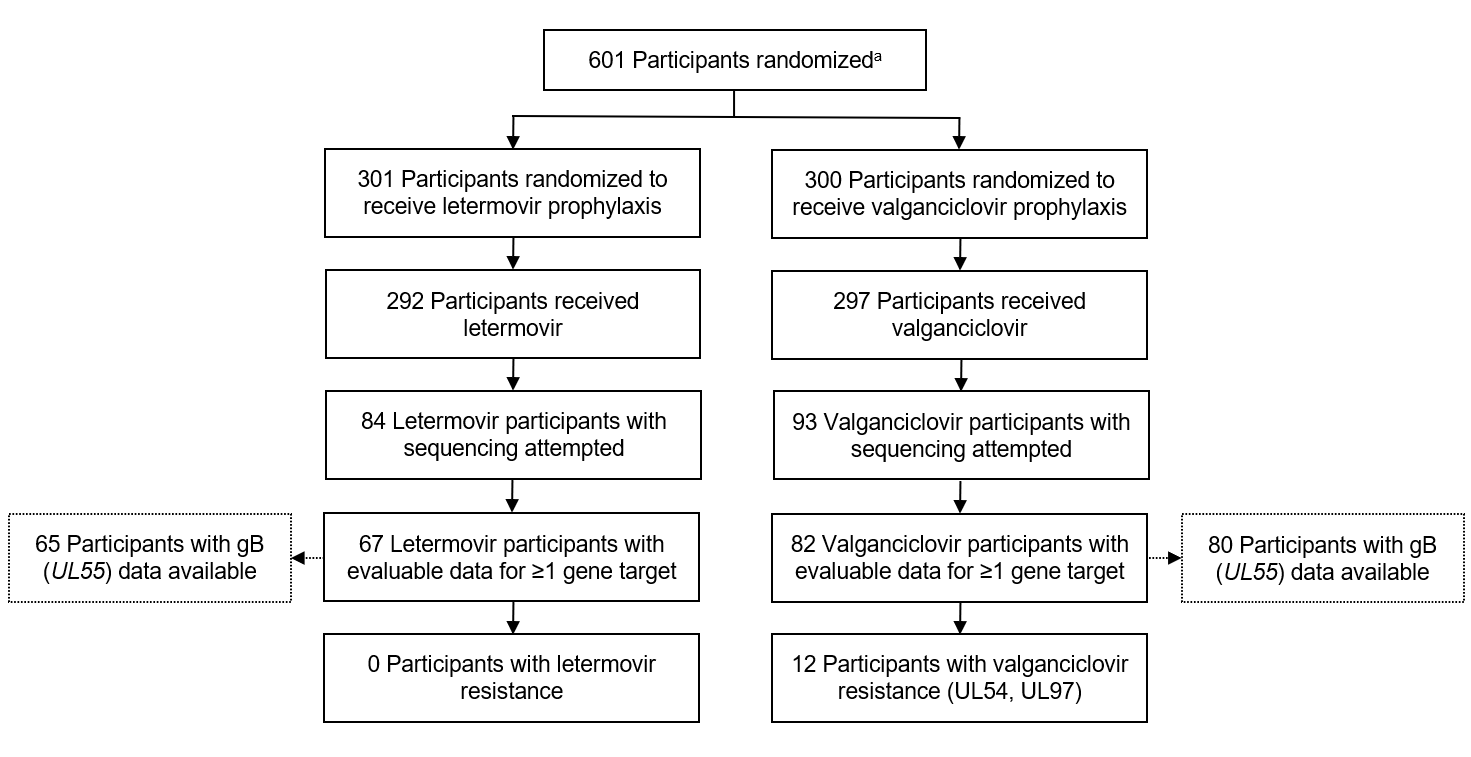


Abbreviation: gB, glycoprotein B.

^a^Stratified by receipt of lymphocyte-depleting induction immunosuppression.
